# A pre-post study testing a lung cancer screening decision aid in primary care

**DOI:** 10.1186/s12911-018-0582-1

**Published:** 2018-01-12

**Authors:** Daniel S. Reuland, Laura Cubillos, Alison T. Brenner, Russell P. Harris, Bailey Minish, Michael P. Pignone

**Affiliations:** 1 Department of General Medicine and Clinical Epidemiology, University of North Carolina School of Medicine, Cecil G Sheps Center for Health Services Research, University of North Carolina at Chapel Hill, 725 Martin Luther King Jr. Blvd, CB 7590, Chapel Hill, NC 27599 USA; 2 Lineberger Comprehensive Cancer Center, Cecil G Sheps Center for Health Services Research, University of North Carolina at Chapel Hill, 725 Martin Luther King Jr. Blvd, CB 7590, Chapel Hill, NC 27599 USA; 3 Department of General Medicine and Clinical Epidemiology, University of North Carolina, School of Medicine, Ambulatory Care Center, University of North Carolina at Chapel Hill, 101 Mason Farm Road, Chapel Hill, NC 27599-7745 USA; 4 Department of Medicine, Dell Medical School, The University of Texas at Austin, 1912 Speedway, Campus Mail Code D2000, Austin, TX 78712 USA

**Keywords:** Cancer screening, Shared decision making, Primary care, Medicare, Pulmonary diseases

## Abstract

**Background:**

The United States Preventive Services Task Force (USPSTF) issued recommendations for older, heavy lifetime smokers to complete annual low-dose computed tomography (LDCT) scans of the chest as screening for lung cancer. The USPSTF recommends and the Centers for Medicare and Medicaid Services require shared decision making using a decision aid for lung cancer screening with annual LDCT. Little is known about how decision aids affect screening knowledge, preferences, and behavior. Thus, we tested a lung cancer screening decision aid video in screening-eligible primary care patients.

**Methods:**

We conducted a single-group study with surveys before and after decision aid viewing and medical record review at 3 months. Participants were active patients of a large US academic primary care practice who were current or former smokers, ages 55–80 years, and eligible for screening based on current screening guidelines. Outcomes assessed pre-post decision aid viewing were screening-related knowledge score (9 items about screening-related harms of false positives and overdiagnosis, likelihood of benefit; score range = 0–9) and preference (preferred screening vs. not). Screening behavior measures, assessed via chart review, included provider visits, screening discussion, LDCT ordering, and LDCT completion within 3 months.

**Results:**

Among 50 participants, knowledge increased from pre- to post-decision aid viewing (mean = 2.6 vs. 5.5, difference = 2.8; 95% CI 2.1, 3.6, *p* < 0.001). Preferences across the overall sample remained similar such that 54% preferred screening at baseline and 50% after viewing; however, 28% of participants changed their preference (to or away from screening) from baseline to after viewing. We assessed screening behavior for 36 participants who had a primary care visit during the 3-month period following enrollment. Eighteen of 36 preferred screening after decision aid viewing. Of these 18, 10 discussed screening, 8 had a test ordered, and 6 completed LDCT. Among the 18 who preferred no screening, 7 discussed screening, 5 had a test ordered, and 4 completed LDCT.

**Conclusions:**

In primary care patients, a lung cancer screening decision aid improved knowledge regarding screening-related benefits and harms. Screening preferences and behavior were heterogeneous.

**Trial registration:**

This study is registered at www.clinicaltrials.gov. NCT03077230 (registered retrospectively,November 22, 2016).

## Background

Lung cancer is the leading cause of cancer death in the United States (US) [1]. The National Lung Screening Trial (NLST) showed that annual low-dose computed tomography (LDCT) can reduce mortality from lung cancer in a high-risk population [2]. However, despite evidence for mortality reduction, LDCT screening can also lead to harms. For example, more than 95% of screen-detected nodules are ultimately determined to be benign (i.e. false positives) after follow-up evaluation, which can be costly and invasive [2]. Further, screening can also lead to the detection and unnecessary treatment of cancers that would not have affected the patient clinically in his/her lifetime (overdiagnosis) [3, 4].

Based on NLST findings and other evidence [5, 6], the US Preventive Services Task Force (USPSTF) found that there was sufficient support for the net benefit of screening to recommend annual screening with LDCT for patients ages 55–80 with 30 or more pack-years smoking history who have smoked in the past 15 years [4]. Because of the tradeoffs between benefits and harms involved, guidelines recommend a thorough process of informed and shared decision-making occur prior to commencing annual screening. [4, 7] However, experts remain concerned that widespread implementation of screening could happen without patients being appropriately informed [8]. These concerns are supported by accumulating evidence that US patients generally overestimate the benefits and are poorly informed about the potential harms of cancer screening [9–14]. These concerns informed a 2015 Centers for Medicare & Medicaid Services (CMS) coverage decision requiring that a “shared decision-making visit” involving the use of a patient decision aid be conducted before screening would be covered for CMS beneficiaries [15, 16].

Decision aids are evidence-based tools designed to facilitate informed and shared decision-making about complex treatments or screening choices [17]. A lung cancer screening decision aid may be a helpful adjunct in conveying the complex information about benefits and harms of screening. However, few lung cancer screening decision aids have been tested and, to our knowledge, none have been studied in a primary care setting, where cancer screening decisions typically take place [18–20]. Moreover, little is known about whether decision aids help screening-eligible patients develop a realistic understanding of the likelihood of benefitting from screening or improve their understanding of important but difficult to understand screening-related harms, such as false positives and overdiagnosis [18, 19].

## Methods

We report the findings from a single-group primary care clinic-based study of a video decision aid on lung cancer screening in a cohort of screening-eligible primary care patients. We had three main study aims: first, to assess the effect of the decision aid on knowledge of the benefits and harms of screening and on screening preferences; second, to describe screening behavior within 3 months of viewing a screening decision aid; and third, to examine relationships between screening knowledge, preferences, and screening test ordering during subsequent primary care encounters.

### Setting and participants

The study setting was an academic internal medicine practice serving approximately 13,000 patients. We identified active patients who were current or former smokers ages 55–80. We excluded patients with lung cancer, cancer treatment with chemotherapy or radiation within 18 months, recent hemoptysis or unexplained weight loss, or any chest CT within 18 months. Study staff reviewed electronic health records (EHR) and further excluded those who clearly did not meet USPSTF smoking history requirements (i.e. fewer than 30 pack-years or quit more than 15 years ago). Primary providers reviewed lists of their potentially eligible patients and excluded those deemed inappropriate for screening based on comorbidities. Approved patients were mailed a recruitment packet containing a study invitation letter and an opt-out card.

Patients then received a recruitment telephone call where an eligibility survey was administered to confirm screening eligibility based on USPSTF guidelines. Eligible patients who agreed to participate were scheduled for a study visit at the clinic. Upon arrival and consent, participants completed a baseline survey, viewed the video decision aid, and completed a follow-up survey. Screening behaviors were assessed via EHR review at 3 months. Participants received a $40 gift card. Data were collected from October 2015–October 2016 and analyzed from October 2016–February 2017.

### Intervention

We previously developed and refined the decision aid based on feedback from screening-eligible patients from the community (*n* = 11) and providers from the academic medical center (n = 11) [21]. The decision aid was designed to meet requirements specified by CMS and relevant standards set by the International Patient Decision Aid Standards Collaboration, and to be accessible to those with low literacy [15, 22]. Written text was read aloud, and technical terms and concepts were explained using narration, graphics, and animations. We pre-tested the decision aid and measures with 10 screening-eligible participants before beginning the pre-post phase of the study.

**Fig. 1:**
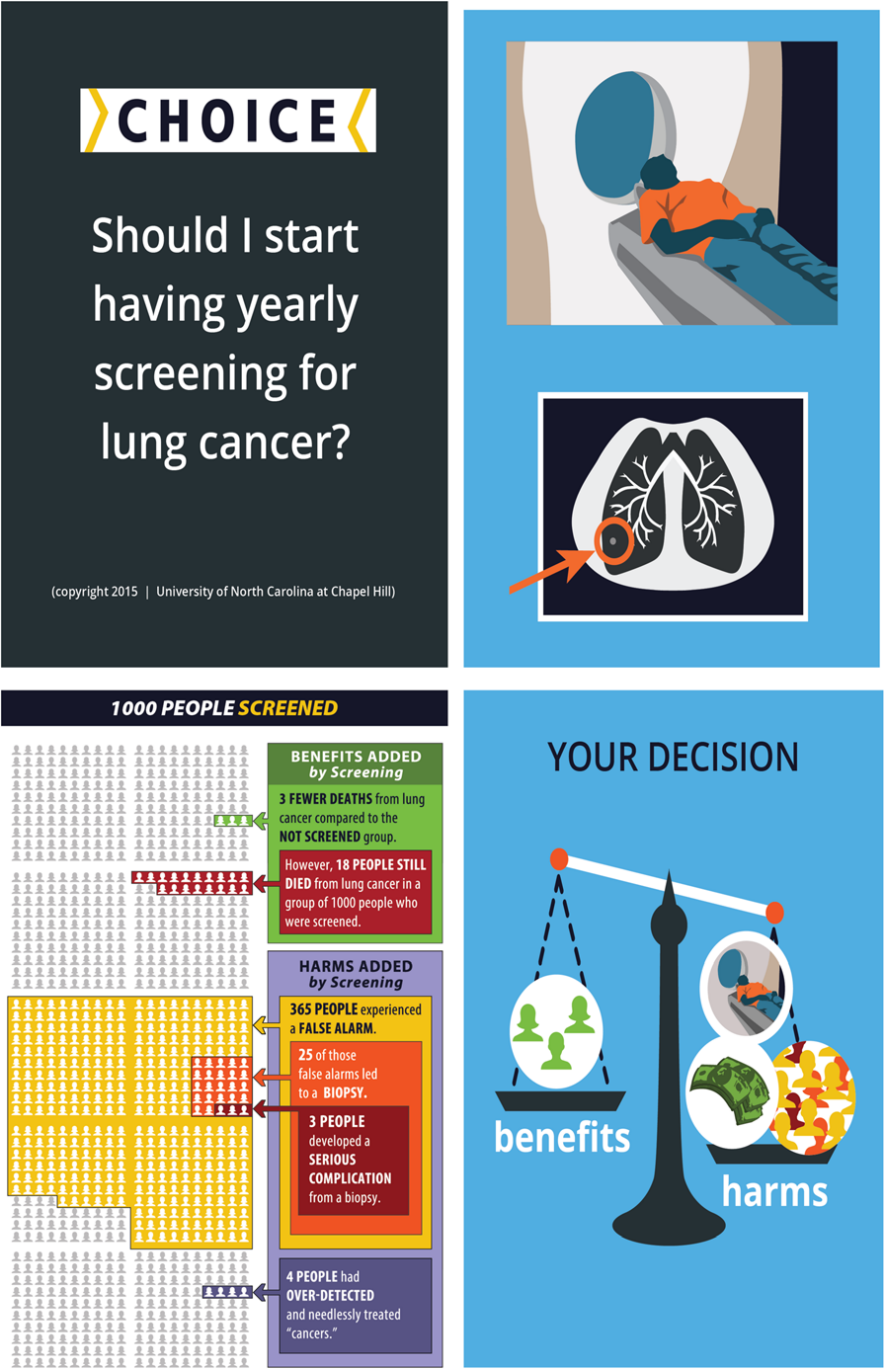
Decision aid screenshots

Content included the rationale for screening, eligibility criteria, a description of the LDCT procedure, and a dynamic icon array (pictogram) that sequentially depicted estimates for benefits and harms of screening among 1000 individuals screened annually for 3 years (Fig. 1). Estimates were based on NLST trial data as presented in materials developed by the National Cancer Institute and the Veterans Health Administration (VHA) [2, 20, 23, 24]. Benefits presented in the pictogram were lung cancer deaths averted (3 per 1000 screened). Harms presented included false positives, need for biopsy that did not find cancer, serious complications from biopsies, and overdiagnosis. Other potential screening harms, presented qualitatively (not in the pictogram) included radiation exposure, anxiety and distress, and costs related to follow-up tests and procedures. The video concluded with an implicit values clarification exercise prompting the viewer to weigh the potential benefits and harms of screening and discuss them with his/her doctor. Participants viewed the 6-min video at the clinic on a tablet computer (available online https://goo.gl/1f7XIY).

### Measures

#### Knowledge

We measured screening-related knowledge before and after decision aid viewing (items shown in Results, Table 2). Knowledge of benefit was assessed using a single item asking about the number of lung cancer deaths averted per 1000 individuals screened. Response options including contiguous estimates ranging from 0 up to 1000. Because our primary goal was to convey the “gist” concept that the number of individuals who benefit is small relative to the number of individuals screened, we treated responses that were within an order of magnitude of the value presented in the decision aid, i.e. up to 30 per 1000, as correct. Knowledge of harms was assessed using seven items adapted from a previously-published overdiagnosis knowledge scale [25] and one item about false positives. Knowledge items were categorized as either correct or incorrect, with “don’t know” responses treated as incorrect. A knowledge score was calculated (0–9 points) by summing the correct answers.

#### Preference

Screening preference was assessed through a single 5-point Likert item adapted from a previous study asking participants how much they agreed with the statement “I plan to have a lung cancer screening test in the near future” (strongly agree to strongly disagree) [26].

#### Clinical screening behavior and LDCT findings

For patients who had a primary care visit, we assessed whether there was EHR documentation of each of the following: screening-related discussion, LDCT order, and LDCT completion. For completed CTs, we also recorded the LungRADS nodule classification based on the radiologist’s report [27].

#### Decision aid acceptability

We assessed decision aid acceptability using a published scale measuring perception of decision aid length, balance, and suitability for decision-making [28].

### Statistical analyses

We characterized the study population with descriptive statistics. We tested for pre-post changes in knowledge using a paired t-test for total score and McNemar’s chi-squared tests for individual items. We dichotomized the preference item, defining “preference for screening” as “agree” or “strongly agree” responses. We calculated the proportions who changed their preference before and after decision aid viewing. We calculated proportions for screening behavior measures and LDCT findings among participants who had a provider visit after viewing the decision aid (and thus an opportunity for screening discussion and test ordering). We examined the relationship between (post-decision aid) knowledge and screening preference using logistic regression controlling for baseline preference. We also calculated the proportion of visits with a “preference concordant” decision, defined as either: preferring screening and having LDCT ordered, OR not preferring screening and not having an LDCT ordered. Analyses were conducted using STATA (Release-13, College Station, TX).

## Results

**Fig. 2:**
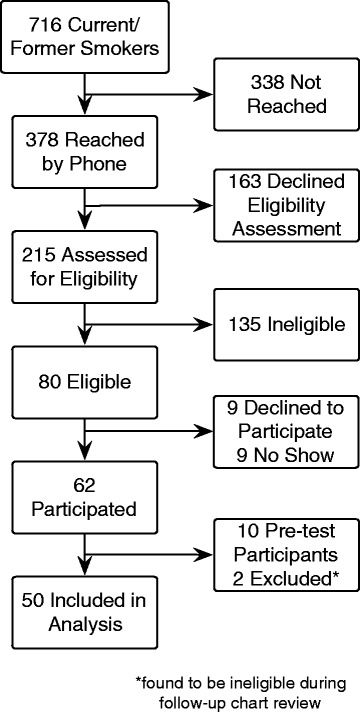
Flowchart of enrollment

Enrollment (see Fig. 2): We mailed 716 recruitment letters and reached 378 patients by telephone. Among 215 patients who completed the initial eligibility assessment, 135 were found ineligible. Common reasons for declining to complete the eligibility assessment included poor health, lack of time, and transportation challenges. Among the 80 patients initially found eligible for the study, 18 either declined participation or did not attend the study visit. Of the 62 (78%) eligible patients who participated, 10 completed the pre-test phase and thus were ineligible to participate in the main pre-post phase. Two additional participants were later found to be ineligible during the follow-up chart review phase of the study (because of recent chest CTs) and were excluded from analysis. The final analytic sample included 50 participants.

Sample characteristics (Table 1) include: mean age of 63 years; 48% female; 58% White; 30% Black; 12% other race; 50% high school education or less; 46% current smokers; average of 52 pack-years smoked; 40% COPD; and 56% had Medicare (alone or with another insurance).

**Table 1:** Participant Characteristics (*n* = 50)

	Average or %
Age	63
Sex (% Female)	48%
Race/Ethnicity	
White	58%
Black	30%
Other	12%
Education	
≤ 12 years	50%
Smoking status (% current)	46%
Pack-years smoked^a^	52
COPD	40%
Insurance Status	
No insurance	8%
Private insurer (only)	28%
Medicare (only)	30%
Medicaid (only)	8%
Medicare, plus other insurer	26%

^a^(average packs per day * years smoked)

### Screening knowledge and preferences

Knowledge score (9-point scale) increased after viewing the decision aid from 2.6 to 5.5 (difference = 2.8; 95% confidence interval [CI] 2.1, 3.6, *p* < 0.001) (Table 2). Before decision aid viewing, the most common response was that 401–700 individuals benefit per 1000 screened. After decision aid viewing, the most common response category was 1–5 per 1000. In the overall sample, 27 (54%) participants preferred screening at baseline and 25 (50%) preferred screening after viewing.

**Table 2:** Changes in knowledge and intent to initiate lung cancer screening before and after viewing the decision aid (*n* = 50)

	Pre	Post	Difference (95% CI), *p*-value
Potential Harms of Screening			
Who do you think is more likely to be diagnosed with lung cancer? *People who are screened for lung cancer.*^a^ *People who are NOT screened for lung cancer*	36%	62%	26% (8%, 44%), *p* < 0.001
ALL lung cancers will eventually cause illness and death if they are not found and treated. *True/False*^a^*/Don’t Know*	6%	54%	48% (31%, 65%), *p* < 0.001
When screening finds lung cancer, doctors can tell whether the cancer will ever cause harm. *True/False*^a^*/Don’t Know*	16%	62%	46% (62%, 30%), *p* < 0.001
Even lung cancers that may not cause any health problems are likely to be treated. *True*^a^*/False/Don’t Know*	66%	80%	14% (4%, 32%), *p* = 0.09
Screening tests lead some people to get cancer treatments that they do not need. *True*^a^*/False/Don’t Know*	18%	76%	58% (40%, 76%), *p* < 0.001
Screening tests find harmless lung cancers about as often as they prevent death from lung cancer. *True*^a^*/False/Don’t Know*	24%	52%	28% (9%, 47%), *p* < 0.001
Which of these 2 statements best describes over-detection from screening? *Screening finds a cancer that would never have caused trouble*^a^ *Screening finds an abnormality but extra tests show it is not cancer*	16%	28%	12% (3%, 27%), *p* = 0.08
An abnormal result from lung cancer screening always means the person has lung cancer. *True/False*^a^*/Don’t Know*	66%	88%	22% (5%, 39%), *p* < 0.001
Chances of Benefitting from Screening			
For the next question, please think about 1000 current and former smokers who are getting screened every year for lung cancer. Out of 1000 people who get a chest CT scan, about how many will have their lives prolonged? *0* *1-5*^a^ *6-10*^a^ *11-30*^a^ *31–100* *101–200* *201–400* *401–700* *701–1000* *Don’t Know*	18%	48%	30% (12%, 48%), *p* < 0.001
Average Knowledge Score (0–9 points)	2.6	5.5	2.8 (2.1,3.6), *p* < 0.001

^a^Correct response(s)

Of the 27 participants who preferred screening pre-decision aid viewing, 8 (30%) changed and did not prefer screening after viewing. Of the 23 participants who did not prefer screening pre-decision aid, 6 (26%) preferred screening post-decision aid.

We observed an inverse relationship between post-decision aid knowledge and screening preference (odds ratio = 0.73; 95% CI 0.54, 0.98; *p* = 0.03). For each point increase in post-decision aid knowledge score there was an estimated 27% reduction in the odds of preferring screening.

### Decision aid acceptability

Most participants (*n* = 48, 96%) reported that the decision aid was “useful in making a decision about getting screened for lung cancer.” Most participants (*n* = 29, 58%) felt that the decision aid was balanced, 16 (32%) indicated that it was slanted toward getting screened, and 5 (10%) indicated that it was slanted toward no screening.

### Screening behavior

**Fig. 3:**
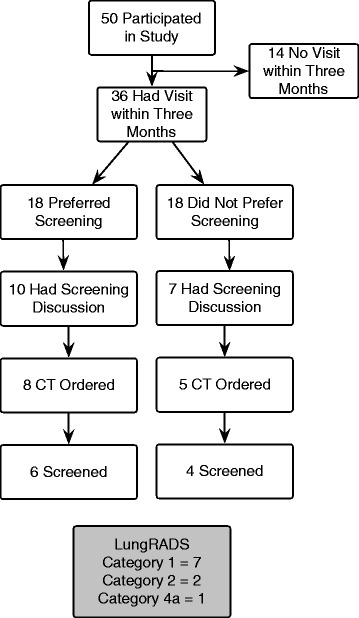
Flowchart of lung cancer screening decisions and behavior among study participants

Thirty-six participants had a clinic visit in the 3 months following study enrollment (Fig. 3), among whom 21 (58%) had concordance between test preference and test ordering. Among 18 participants preferring screening after decision aid viewing, 10 (56%) discussed screening, 8 (44%) had a test ordered, and 6 (33%) completed LDCT. Among the 18 not preferring screening, 7 (39%) discussed screening, 5 (28%) had a test ordered, and 4 (22%) completed LDCT. Discordance was greatest for the 18 participants who indicated they preferred screening, of whom 10 did not subsequently have an LDCT ordered. Notably, in 8 of these 10 cases, there was no apparent screening discussion with the provider.

### LDCT findings

Among the 10 completed LDCTs, 7 were LungRADS category 1 (normal result) and 2 were category 2 (small nodules, benign appearance). One was category 4a (suspicious findings); this participant preferred screening in the study and the recommended 3-month follow-up scan showed resolution of the nodule.

## Discussion

We report findings from testing a lung cancer screening decision aid in 50 primary care patients. We found that decision aid viewing was associated with greater knowledge of the benefits and harms of screening. At baseline, we found that participants tended to greatly overestimate the chances of benefitting from screening. After viewing, participants tended to have a more realistic understanding of the chances of benefitting. We also found that decision aid viewing led to improved understanding of two important but conceptually complex screening-related harms: false positives and overdiagnosis. To our knowledge, this study is the first to demonstrate that viewing a decision aid can help attenuate biased perceptions about the benefits and harms of lung cancer screening.

Our study complements and extends a limited body of empiric evidence about lung cancer screening decision aids. Previous studies were conducted in populations recruited from the community [19], or referral settings such as a tobacco cessation clinic [18] or a dedicated, sub-specialty screening program [20]. Our study was conducted in a primary care setting and allowed providers to exclude patients they believed to be poor screening candidates based on co-morbidity concerns. Thus, despite being relatively small, our study mimicked a systematic, practice-based approach to screening. Furthermore, two of three prior studies tested lung cancer screening decision aids in mixed populations that included relatively small numbers of screening eligible patients (*n* = 14 and *n* = 11, respectively) [18, 19]. All participants in our study were eligible for screening based on current USPSTF guidelines.

We found that baseline (pre-decision aid) screening preferences were heterogeneous, with roughly half of participants falling into each of our two preference groups (preferring screening vs. not). Although decision aid viewing was not associated with net changes in the proportions in each of these preference groups in the overall study sample, about one-quarter of participants in each group actually changed their screening preference after decision aid viewing (in opposite directions). Our findings suggest that we cannot assume screening-eligible primary care patients will be uniformly inclined toward or away from screening. Moreover, our results also suggest that a non-trivial proportion of primary care patients may change their preferences as they become more informed about screening.

Our results are consistent with the findings of Kinsinger et al.’s VHA pragmatic screening demonstration pilot [29] in that we observed heterogeneity in screening behavior. Among participants who preferred screening and saw a provider within 3 months of decision aid viewing, about half discussed LDCT scanning with their provider and about a third completed an LDCT. We also found that some participants who did not prefer screening after decision aid viewing ended up completing an LDCT. Kinsinger et al. similarly found that 50% of eligible patients completed screening [29]. While the VHA demonstration program developed and utilized paper-based decision support materials (from which we adapted our video decision aid), no data are available regarding the manner and extent to which they were used.

Our findings that 50% of patients preferred screening post-decision aid and 28% completed screening (among patients with post-decision aid primary care encounters), contrasts with findings from what is, to our knowledge, the only other study to assess screening completion in individuals receiving a lung cancer screening decision aid. That study, conducted by Mazzone et al. in patients attending a tertiary screening program, found that 95% of participants completed screening after undergoing a shared decision making visit [20]. We hypothesize that the overwhelming majority of patients who attend a dedicated lung cancer screening program assume that the purpose of referral to such a program is to complete screening, rather than to decide about screening. When considering Mazzone’s findings alongside our results, it suggests that the time frame during which screening-eligible patients actually make decisions about screening is before they attend a dedicated screening program. Tertiary lung cancer screening programs are now being implemented in the US, and many claim to be able to conduct shared decision making. Our findings add to the discourse regarding the appropriate implementation context for medical decision making about lung cancer screening.

We found that the more knowledge participants had about benefits and harms of screening, the less likely they were to prefer screening. The fact that we did not observe differences in screening behavior suggests the need for additional research aimed at understanding how we can help ensure that patients receive care that is consistent with their preferences. This will likely require interventions beyond providing patient decision aids.

Our study examined decision aid effects on knowledge about two important harms of screening: overdiagnosis and false positives. We chose these outcomes because: 1) overdiagnosis and false positive tests can cause substantial harms in screened populations; 2) CMS explicitly requires that information about overdiagnosis and false positives be included in lung cancer screening decision aids; and 3) these harms may not be recognized or understood by patients making medical decisions about cancer screening [9, 15, 25]. Other knowledge domains are also probably relevant to decisions about lung cancer screening [8]. Further research is needed to assess the validity of measures of decision-relevant knowledge and to better understand knowledge thresholds at which patients may be considered adequately informed to make medical decisions about cancer screening.

We found relatively low concordance (58%) between (post-decision aid) preferences and LDCT ordering during subsequent provider visits. It appears that a major driver of discordance was that many patients who preferred screening and did not have an LDCT ordered did not actually have an opportunity to discuss screening with their provider. This finding points to an important problem in implementation of patient decision support in health care. Decision aids are intended not to replace but to inform discussions between patients and providers about medical decisions [30, 31]. However, competing demands and lack of adequate provider time to deliver preventive services are important barriers to effective communication and decision-making [16]. Further, lung cancer screening is especially complex given both the risk for lung cancer in this population and the chances of serious complications as a consequence of screening. Thus, further research is needed to understand how best to structure lung cancer screening decision support interventions in the primary care medical home to ensure that there is adequate time for patients and providers to discuss and deliberate. Moreover, more research is needed to understand the role that domains other than knowledge and stated preferences play in the complex picture of lung cancer screening behavior.

### Limitations

Our study has limitations. First, because of the one-group, pre-post study design we were unable to compare the effects of the decision aid with usual care. Second, this was a single site study and many patients declined initial eligibility assessment. Additionally, patients received an incentive to participate and were required to attend a separate study visit, both of which can affect participant behavior and sample representativeness. Thus, the degree to which the findings are generalizable to other screening-eligible populations is unclear. Nevertheless, our participants were similar to NLST participants in terms of average age (63 in our study, 62 in NLST), pack-years smoked (52 vs. 56, respectively), percent current smokers (46% vs. 48%, respectively), and percent female (48% vs 41%, respectively) [32]. Our sample reflects a more disadvantaged population than was studied in the NLST in that they were less likely to be white (58% vs 91%, respectively) and less likely to have received education beyond high school (50% vs 70%, respectively) [33]. Third, knowledge was assessed immediately following completion of the decision aid and may not reflect long-term retention of lung cancer screening information. Finally, our behavior assessments were relatively crude in that we did not differentiate between chest CT scans discussed and ordered for diagnostic reasons (i.e. to evaluate symptoms) vs. for true screening.

Another consideration is that the decision aid we tested was not targeted to lung cancer risk. Ideally, information about benefits and harms of screening would be tailored to the patient’s individual lung cancer risk, as occurs in the decision aid produced at the University of Michigan [33]. However, brief video decision aids such as ours or the one developed by Volk and colleagues [18] do not require high-level reading capability, entry of patient-specific data, or other interaction by the patient. Such a format offers potential advantages in terms of implementation, and may be better suited for low-literacy populations. Studies are needed to examine tradeoffs associated with using simpler versus more complex, tailored decision aids for lung cancer screening, particularly given the prevalence of low education and low literacy in the US screening-eligible population.

## Conclusions

We found that, among primary care patients who are eligible for lung cancer screening based on USPSTF guidelines, viewing a lung cancer screening decision aid improved screening-related knowledge. It gave patients a more realistic perception of the likelihood of benefit. It also gave them a greater understanding of the nature of important screening-related harms. Our findings, although preliminary, suggest that a non-trivial proportion of screening-eligible patients may change their screening preferences after decision aid viewing. In contrast to findings in a referral population at a tertiary, sub-specialty screening program, screening preferences and behaviors among screening-eligible patients in a primary care population appear to be heterogeneous.

## Abbreviations

CI: Confidence Interval
CMS: Center for Medicare and Medicaid Services
EHR: Electronic Health Record
LDCT or CT: Low-Dose Computed Tomography or Computed Tomography
NLST: National Lung Screening Trial
US: United States
USPSTF: United States Preventive Services Task Force
VHA: Veterans Health Administration
